# Supplementary material for: Ethylene modulates cell wall mechanics for root responses to compaction
Source: Nature. 2025 Nov 26;649(8097):685–92. doi: 10.1038/s41586-025-09765-7 (PMC12804079; doi:10.1038/s41586-025-09765-7)
Supplement: Supplementary file 1 — Reporting Summary [file 41586_2025_9765_MOESM1_ESM.pdf]

nature portfolio

Corresponding author(s): Wanqi Liang, Bipin Pandey, Staffan Persson

Last updated by author(s): Oct 10, 2025

Reporting Summary

Nature Portfolio wishes to improve the reproducibility of the work that we publish. This form provides structure for consistency and transparency in reporting. For further information on Nature Portfolio policies, see our Editorial Policies and the Editorial Policy Checklist.

Statistics

For all statistical analyses, confirm that the following items are present in the figure legend, table legend, main text, or Methods section.

n/a	Confirmed	
☐	☒	The exact sample size (*n*) for each experimental group/condition, given as a discrete number and unit of measurement
☐	☒	A statement on whether measurements were taken from distinct samples or whether the same sample was measured repeatedly
☐	☒	The statistical test(s) used AND whether they are one- or two-sided<br>*Only common tests should be described solely by name; describe more complex techniques in the Methods section.*
☒	☐	A description of all covariates tested
☐	☒	A description of any assumptions or corrections, such as tests of normality and adjustment for multiple comparisons
☐	☒	A full description of the statistical parameters including central tendency (e.g. means) or other basic estimates (e.g. regression coefficient) AND variation (e.g. standard deviation) or associated estimates of uncertainty (e.g. confidence intervals)
☐	☒	For null hypothesis testing, the test statistic (e.g. *F*, *t*, *r*) with confidence intervals, effect sizes, degrees of freedom and *P* value noted<br>*Give P values as exact values whenever suitable.*
☒	☐	For Bayesian analysis, information on the choice of priors and Markov chain Monte Carlo settings
☒	☐	For hierarchical and complex designs, identification of the appropriate level for tests and full reporting of outcomes
☒	☐	Estimates of effect sizes (e.g. Cohen's *d*, Pearson's *r*), indicating how they were calculated

Our web collection on statistics for biologists contains articles on many of the points above.

Software and code

Policy information about availability of computer code

Data collection	CT scan was imaged using a GE Phoenix v|tome|x M 240 kV X-ray tomography system;<br>Root sections were taken by a Leica Vibratome (VT 1000 S), and imaged with a Leica TCS SP5 confocal microscope;<br>GUS images were taken by a a Nikon H600L light microscope (Tokyo, Japan);<br>TEM images were taken by a JEM-1230 transmission electron microscope (JEOL) at 80 kV;<br>AFM results were taken by a Dimension ICON (Bruker Nano).
Data analysis	The co-expression network of CESAs analyzed via https://conekt.sbs.ntu.edu.sg/;<br>The CT three-dimensional image reconstruction was carried out using Datos|REC software (GE Inspection Technologies, Wunstorf, Germany);<br>Statistical analyses of all graphs were performed using GraphPad Prism (Version 9.2.0) (https://www.graphpad.com/);<br>Phylogenetic analysis data of CESA homologs was analyzed using the MEGA software (Version 5.0);<br>Microscopy images were analyzed by ImageJ2 (Version 2.14.0/1.54f);<br>AFM results were analyzed by the NanoScope Analysis 9.4 software.

For manuscripts utilizing custom algorithms or software that are central to the research but not yet described in published literature, software must be made available to editors and reviewers. We strongly encourage code deposition in a community repository (e.g. GitHub). See the Nature Portfolio guidelines for submitting code & software for further information.

Data

Policy information about availability of data

All manuscripts must include a data availability statement. This statement should provide the following information, where applicable:

- Accession codes, unique identifiers, or web links for publicly available datasets
- A description of any restrictions on data availability
- For clinical datasets or third party data, please ensure that the statement adheres to our policy

All information supporting the conclusions are provided with the paper. Primers used in this study is provided in Supplementary Table 1. Gene accession number information is available in Supplementary Table 2. Yeast one-hybrid screening results is provided in Supplementary Data 1, and raw data for EMSA (gel) is provided in Supplementary Data 2. Source Data for Main Figures and Extended Data Figures are also provided with this paper.

Research involving human participants, their data, or biological material

Policy information about studies with human participants or human data. See also policy information about sex, gender (identity/presentation), and sexual orientation and race, ethnicity and racism.

Reporting on sex and gender	N/A
Reporting on race, ethnicity, or other socially relevant groupings	N/A
Population characteristics	N/A
Recruitment	N/A
Ethics oversight	N/A

Note that full information on the approval of the study protocol must also be provided in the manuscript.

Field-specific reporting

Please select the one below that is the best fit for your research. If you are not sure, read the appropriate sections before making your selection.

☒ Life sciences ☐ Behavioural & social sciences ☐ Ecological, evolutionary & environmental sciences

For a reference copy of the document with all sections, see nature.com/documents/nr-reporting-summary-flat.pdf

Life sciences study design

All studies must disclose on these points even when the disclosure is negative.

Sample size	Sample sizes were determined based on standard practices in plant biology research and experimental objectives. For example, for root cortical diameter measurements, approx. 40 cells from 5 sections per genotype were imaged per biological replicate, with three biological replicates performed. For AFM analysis, 15 measurement points were tested per genotype across four biological replicates. For TEM imaging, two sections were examined per biological replicate, with five biological replicates performed per genotype. These are typical sample sizes and repetitions compared to similar experimental analyses.
Data exclusions	We did not exclude any data from the results.
Replication	Each experiment was repeated independently from twice to five replicates, with all replicates showing consistent trends.
Randomization	All the experiments were performed without prior knowledge of the final outcome, and therefore randomization was not applied.
Blinding	All the experiments were performed without prior knowledge of the final outcome, and therefore blinding was not applied on most of our analyses. However, we did perform blind experiments on the cell wall thickness analyses as these are sampling error prone.

Reporting for specific materials, systems and methods

We require information from authors about some types of materials, experimental systems and methods used in many studies. Here, indicate whether each material, system or method listed is relevant to your study. If you are not sure if a list item applies to your research, read the appropriate section before selecting a response.

Materials & experimental systems

n/a	Involved in the study
☐	☒ Antibodies
☒	☐ Eukaryotic cell lines
☒	☐ Palaeontology and archaeology
☒	☐ Animals and other organisms
☒	☐ Clinical data
☒	☐ Dual use research of concern
☐	☒ Plants

Methods

n/a	Involved in the study
☒	☐ ChIP-seq
☒	☐ Flow cytometry
☒	☐ MRI-based neuroimaging

Antibodies

Antibodies used

Commercial antibodies used: α-GFP (G1544, Sigma, 1:5000 dilution), α-His (M20003, Abmart, 1:2000 dilution), Goat Anti-Mouse IgG HRP (M21001, Abmart, 1:5000 dilution), Goat Anti-Rabbit IgG Antibody (AP132, sigma, 1:5000 dilution).

Validation

Validation statements relevant citations of commercial antibodies available from manufactures
α-GFP (G1544, Sigma): https://www.sigmaaldrich.com/US/en/product/sigma/g1544
anti-His (M20001, Abmart): http://www.ab-mart.com.cn/page.aspx?node=%2059%20&id=%20959
Goat Anti-Mouse IgG HRP (M21001, Abmart): https://www.ab-mart.com.cn/page.aspx?node=%2062%20&id=%20960
Goat Anti-Rabbit IgG Antibody (AP132, Sigma): https://www.sigmaaldrich.com/US/en/product/mm/ap132

Dual use research of concern

Policy information about dual use research of concern

Hazards

Could the accidental, deliberate or reckless misuse of agents or technologies generated in the work, or the application of information presented in the manuscript, pose a threat to:

No	Yes
☒	☐ Public health
☒	☐ National security
☒	☐ Crops and/or livestock
☒	☐ Ecosystems
☒	☐ Any other significant area

Experiments of concern

Does the work involve any of these experiments of concern:

No	Yes
☒	☐ Demonstrate how to render a vaccine ineffective
☒	☐ Confer resistance to therapeutically useful antibiotics or antiviral agents
☒	☐ Enhance the virulence of a pathogen or render a nonpathogen virulent
☒	☐ Increase transmissibility of a pathogen
☒	☐ Alter the host range of a pathogen
☒	☐ Enable evasion of diagnostic/detection modalities
☒	☐ Enable the weaponization of a biological agent or toxin
☒	☐ Any other potentially harmful combination of experiments and agents

Plants

Seed stocks	arf1, cesa6, OE-ARF1, NG-CESA6, arf1cesa6 were obtained in lab. Nip, ein2, eil1eil2 were obtained from Rongfeng Huang's lab. All seeds were grown and collected at the paddy field of Shanghai Jiao Tong University, under natural conditions from June to October.
Novel plant genotypes	arf1, cesa6 and arf1cesa6 were obtained by CRISPR/Cas9, OE-ARF1, NG-CESA6 were transformed into wild type (9522) or cesa6 mutant background by the staff in lab.
Authentication	The genotype of each mutant was obtained from DNA extracted from a single plant, amplified by PCR, and verified by sequencing. The mutant are stable plant lines in which Cas9 was out-crossed. The RNA extracted from each over-expressed plant was verified by qPCR. These operations are outlined in the method.
