# Supplementary figures and images for: Ethylene modulates cell wall mechanics for root responses to compaction
Source: Nature. 2025 Nov 26;649(8097):685–92. doi: 10.1038/s41586-025-09765-7 (PMC12804079; doi:10.1038/s41586-025-09765-7)

Supplementary Data 2. EMSA raw gel figure in main Fig. 2d

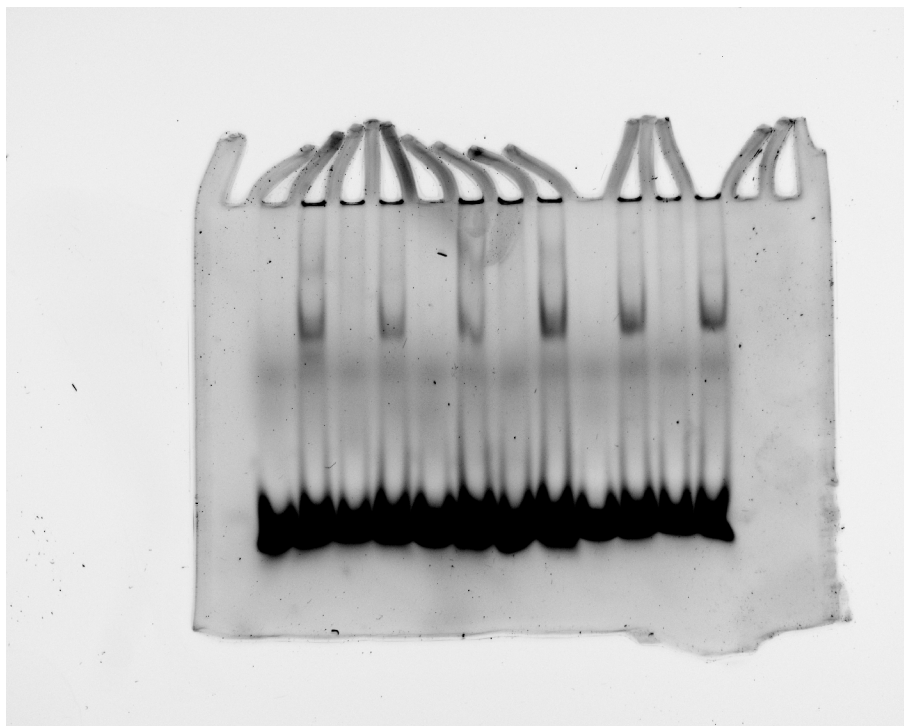

Supplement: Supplementary file 5 — EMSA raw gel figure in main Fig. 2d. [file 41586_2025_9765_MOESM5_ESM.pdf]
